# Resection of the Lungs

**Published:** 1890-07-05

**Authors:** 


### RESECTION OF THE LUNGS.

In our issue of May 3rd we alluded, under the heading of "Pneumotomy," to a case reported in an evening daily of the alleged removal of a tuberculous lung by Professor Tillmanns, of Leipzic. We were not unnaturally incredulous, and concluded that it was merely the evacuation of a pneumonic abscess, an operation several cases of which we had already described. We have, however, now before us the full report of this case as given by Dr. Tillmanns at the Congress of the German Surgical Society at Berlin in April, and find that our lay contemporary had in no way exaggerated the nature, audacity, or success of the operation. In 1888 the patient has already been for two years confined to his bed, and was greatly emaciated. There was extensive tuberculosis of the left lung and pleura, together with emphysema. The lung was quite incapable of function, and the chest wall anteriorly riddled with numerous fistulæ. Fortunately, and somewhat remarkably, the right lung was still sound. Tillmann actually removed the whole left anterior wall of the thorax and scraped the pulmonary pleura and lung till no tubercular nodules or deposits remained visible. Some days later, he transplanted so much of the skin as he had been able to save on to the surface of the collapsed lung, pericardium, &c., adhesion followed, and the patient made a good recovery. The left lung was of course useless, but tuberculization was arrested, and the right lung has remained free from disease. The man is still in as good health as is possible under the circumstances, and has been able to follow his business for two years. The limitation to one side of tuberculosis involving the whole of the pulmonary and costal pleura, which alone rendered the operation practicable, was almost unique, but so is the operation, on the conception and successful execution of which the professor is none the less to be congratulated, and few, if any, will have the opportunity or the courage to emulate his achievement.

